# Pretreatment for biorefineries: a review of common methods for efficient utilisation of lignocellulosic materials

**DOI:** 10.1186/s13068-019-1634-1

**Published:** 2019-12-23

**Authors:** Mats Galbe, Ola Wallberg

**Affiliations:** Department of Chemical Engineering, Lund University, P.O. Box 124, 221 00 Lund, Sweden

**Keywords:** Review, Biomass, Pretreatment, Lignocellulosic materials, Biorefinery, Fractionation

## Abstract

The implementation of biorefineries based on lignocellulosic materials as an alternative to fossil-based refineries calls for efficient methods for fractionation and recovery of the products. The focus for the biorefinery concept for utilisation of biomass has shifted, from design of more or less energy-driven biorefineries, to much more versatile facilities where chemicals and energy carriers can be produced. The sugar-based biorefinery platform requires pretreatment of lignocellulosic materials, which can be very recalcitrant, to improve further processing through enzymatic hydrolysis, and for other downstream unit operations. This review summarises the development in the field of pretreatment (and to some extent, of fractionation) of various lignocellulosic materials. The number of publications indicates that biomass pretreatment plays a very important role for the biorefinery concept to be realised in full scale. The traditional pretreatment methods, for example, steam pretreatment (explosion), organosolv and hydrothermal treatment are covered in the review. In addition, the rapidly increasing interest for chemical treatment employing ionic liquids and deep-eutectic solvents are discussed and reviewed. It can be concluded that the huge variation of lignocellulosic materials makes it difficult to find a general process design for a biorefinery. Therefore, it is difficult to define “the best pretreatment” method. In the end, this depends on the proposed application, and any recommendation of a suitable pretreatment method must be based on a thorough techno-economic evaluation.

## Background

Lignocellulosic biomass has been suggested as an important part of a sustainable society for a few decades. It is viewed as an alternative to fossil carbon sources, such as oil, natural gas and coal. An example is the global biofuel production, which in 2016 was estimated to be 137 billion L (3.3 EJ) [1]. Another field of application is for production of polymers, fertilisers, etc., but the transportation sector is the main consumer of energy-related products. The annual global production of biomass is estimated to be about 4500 EJ of solar energy that is captured per year [2]. Another estimation presents the global annual production to be 1 × 10^11^ tons [3]. About 5%, or 225 EJ/year, is equivalent to almost half of the global energy demand at present. This estimated demand (225 EJ/year) is in line with results from other studies. Berndes et al. [4] gathered data from 17 studies in a review and concluded the contribution from biomass to be between 100 EJ/year and above 400 EJ/year in 2050. The main reasons for the quite large difference are two major uncertainties: availability of land and yield in crop production. In the same publication, the assessed plantation capacity is estimated to yield a sustainable availability between 50 and 240 EJ/year. Thus, it is very difficult to make accurate estimations of the actual amount available. Although global bio-based chemical and polymer production is estimated to be around 50 million tonnes, the historic low price of fossil feedstock together with optimised production processes have restricted commercial production of bio-based products [5]. Some processes for production of chemicals have already reached the required technical maturity to be produced through biological routes. For example, most lactic acid production takes place through a biological route, while the classical chemical route is less important [6].

### The biorefinery concept and full-scale operation

**Fig. 1:**
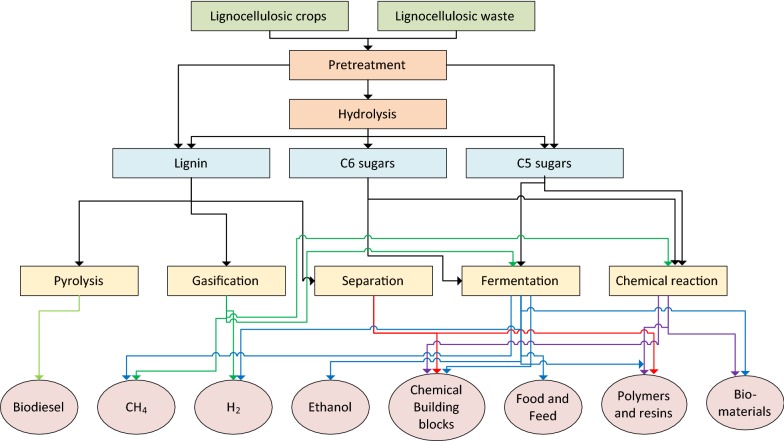
A schematic representation of a biorefinery for production of energy carriers and chemicals

A biorefinery can be defined as the renewable equivalent of a petroleum refinery, the main difference being the raw material. In the biorefinery, biomass can be converted into a wide range of chemicals and energy carriers, and it can also contribute to the development of circular economy; this concept is based on the model that lignocellulosic materials, which were used to generate bio-based products can be recovered (to a certain degree), and be recovered and recycled [7]. The International Energy Agency Bioenergy Task 42 defines biorefining as “the sustainable processing of biomass into a spectrum of marketable bio-based products (chemicals, materials) and bioenergy (biofuels, power, heat)” [8]. However, the different types of raw material constitute a tremendous challenge when a large-scale production facility is considered. In Fig. 1, a schematic overview of a potential biorefinery is shown.

Some important considerations have been suggested for the biorefinery concept to become a path forward towards a less fossil-dependent society. The development of biorefineries is a vital key for integration of food, feed, chemicals, fuels and energy production in the future. Combinations of physical and biotechnological processes for production of proteins, but also for platform chemicals such as lactic acid will be of importance in the future [9, 10]. Biomass can mitigate, to some extent, the high atmospheric levels of carbon dioxide by replacing fossil fuels; in addition, in many countries around the world, the concept may be important to secure domestic energy carriers and the supply of chemicals. Another aspect is the possibility for erection of new industries in rural areas, where it is likely that many of the large biorefineries will be located [11]. Most lignocellulosic feedstock is generally much more complex and recalcitrant than the currently used starchy materials in, e.g. the ethanol industry, which poses a challenge [12]. Nevertheless, a number of demo-scale plants have been put in operation during the last 10–15 years. Several companies have evaluated ethanol production through transformation of cellulose to second-generation sugar products for conversion to ethanol and other chemicals [5]. An easily accessible source (for a quick overview of various facilities worldwide for the production of advanced liquid and gaseous biofuels intended for transport purposes) is maintained by the International Energy Agency (IEA Bioenergy, Task 39) [13], which shows that there is a great interest in energy carriers from biomass. Some examples are POET-DSM, who operates a demo-scale plant at TRL 8, in Emmetsburg, Iowa, USA with a capacity of 75,000 t/year of ethanol. In Finland, the company North European Oil Trade Oy (formerly ST1) is operating a demo plant at TRL 6-7 in Kajaani for production of cellulosic ethanol at a capacity of about 8000 t/year for demonstration of their conversion technology called “Cellulonix”. Similar projects can be found in Brazil (Gran Bio, Bioflex 1), as well as in China (Henan 1), the NREL Integrated Biorefinery Research Facility (IBRF) in Golden (Co, USA), and in several other locations all over the world. However, many earlier projects are now idle or changing focus. An example is a demo plant operated by DuPont in Vonore, TN, US, which is currently idle as DuPont is refocusing on enzyme production. The data gathered from demo-scale plants are extremely important, since they have resulted in large sets of engineering design information, which are necessary for the full-scale design of future biorefineries. However, since most of the recorded data are proprietary information, it is not likely to be accessible to the general engineering or science community. In addition, many pulp mills can be considered, to some extent, to be operated as biorefineries. Today, not only pulp is a valuable product, but also tall oil from the kraft process, which can be transformed to a diesel-like liquid fuel [14], soil conditioners from organic materials [15], fibres for textile production [16], lignin [17], vanillin, microfibrillar cellulose [18], and other valuable products, which in the future may be adding to the revenue from pulp production.

A number of challenges have been identified for implementation of successful future biorefineries, which are summarised in Table 1. Sanford et al. [19] argues that scaling up a successful small-scale operation to a large-scale biorefinery is the first step towards a significant capital investment. Therefore, a very strong economic incentive must be present to justify the investment. In addition, investors also recognise the low return on invested capital and an unstable future situation, as the laws regarding biofuels and biochemicals are not yet long-term stable, which means that the required financial ground is not yet fully prepared for large investments. They also conclude that many biotechnology companies, who have been running a successful early-stage process, have suffered severe problems due to the challenges associated with scale-up and effects from delays in construction, testing and operation. In some cases, capital costs have been cut by retrofitting existing plants. A number of bottlenecks and possible solutions for commercialisation have been identified by Chandel et al. [5]. Some of the important factors are: (1) biomass availability, which includes all-year round supply of lignocellulosic biomass at competitive cost, and access to biomass of similar kind to be able to run a biorefinery within a narrow range of operating conditions. (2) All logistics and a fully working supply chain must be available, e.g. transports of low-density materials (straw, bagasse, etc.), as well as storage facilities for continuous operation to be possible. Feedstock handling and transportation are the most prominent costs associated with the biorefinery concept. In the case of biofuel production, as much as 40–60% of the full-scale production costs can be attributed to feedstock [20, 21]. Tao et al. performed process and techno-economic analysis of six biomass pretreatment processes [22]. The overall ethanol production, total capital investment and minimum ethanol-selling price were compared for the selected methods. The result indicates that there was no large difference in economic performance except if a process was operated in a way, which resulted in low glucose and ethanol yields. In addition to the factors previously discussed, other important challenges to overcome include process mechanisation; transfer of small-scale laboratory data to engineering design data; and the lack of technical maturity in 2G lignocellulosic technology, such as feeding issues with high-solids suspension to a high-pressure reactor.

**Table 1:** A selection of challenges for successful implementation of biorefineries

Problem	Challenge
Scale-up to industrial scale	Requires significant capital investment Requires strong financial incentive Investors find too low return on investment
Future situation unclear	Laws and regulations not clear
Construction and design	Delays in erection of plant Testing of equipment
Biomass availability	All-year round supply of suitable materials Possibility to run on more than one material
Logistics and supply	Storage and transportation must be reliable
Data for process design	Transfer of smaller-scale data to industrial scale
Maturity of a process	Handling at high pressures and feeding, e.g. for 2G plants of ethanol causes production stop

### Platform chemicals

A number of chemicals—platform chemicals—are more important in the biorefinery concept than other products or intermediate compounds. The reason for their importance is that they can to be used as building blocks for production of high-value chemicals. These often constitute chemicals having multi-functional groups that can be converted to other groups of useful molecules. An important feature of a platform chemical is its applicability for production of a multitude of compounds, such as various forms of plastics, binders, fibres, energy carriers, etc. In a joint report from Pacific Northwest National Laboratory (PNNL), National Renewable Energy Laboratory (NREL) and the Office of Energy Efficiency & Renewable Energy (EERE), [23] 12 sugar-based building blocks are targeted as the most important. These include 1,4-diacids (succinic, fumaric and malic), 2,5-furan dicarboxylic acid, 3-hydroxy propionic acid, aspartic acid, glucaric acid, glutamic acid, itaconic acid, levulinic acid, 3-hydroxybutyrolactone, glycerol, sorbitol, and xylitol/arabinitol. A couple of these are considered to be of more interest in terms of their commercial usefulness: succinic, fumaric, and malic acid, since they today form the basis for many high-value replacement products.

### Techno-economic estimations and life-cycle analysis (LCA) for biorefineries

Pretreatment is one of the key unit operations in a biorefinery. Thus, it is critical to evaluate the pretreatment step in conjunction with all related equipment and process solutions in a proposed biorefinery. Life-cycle analysis and techno-economic evaluations are important tools for finding suitable and sustainable production methods. It is not possible to evaluate stand-alone pretreatment systems, since they are always part of a larger processing concept for producing fuels and chemicals. These production processes can, and in most cases do, result in a pallet of products. Some of the pretreatment processes are specialised towards certain products, while others are more generic for the sugar platform. Regardless of which, since the pretreatment step is integrated with other processing steps it is difficult to predict the data for a life-cycle analysis based on a stand-alone pretreatment process only. The primary energy for the pretreatment may be supplied by a downstream processing step at a higher temperature level, and the pretreatment process may supply secondary heat the rest of the process. Both of these alternatives would make the pretreatment process a net zero user of energy, which in an LCA analysis of a pretreatment system is one of the major parts, since one of the major LCA “costs” for a biorefinery is the primary energy supply. If a by-product from the biorefinery is utilised, or if an external energy supply is used, this will strongly influence the LCA performance. It is therefore difficult to make an LCA comparison between different pretreatment processes without taking into account the surrounding processes and the integration with these.

However, in terms of techno-economic analysis of pretreatment, it can be performed to some extent for some of the more mature processes in terms of capital and expenditure costs (CAPEX) for the processing equipment. For less developed processes, the available data are more scarce and unreliable. The comparison between processes at different maturity would likely result in a skewed view on the performance of the different processes and would provide little general information. As with LCA analysis, a techno-economic analysis therefore need to be performed on a case-by-case basis for a certain production process integrated with the pretreatment process. Several estimations on techno-economic analysis and LCA have been published over the years. Aden and Foust [24], as well as Humbird et al. [25] performed a techno-economic analysis on a process based on dilute-acid pretreatment of corn stover followed by enzymatic hydrolysis and fermentation. In the studies, it is concluded that techno-economic analysis plays an important role in process development for targeting of technical as well as economic hurdles, which must be studied for production to be successful. In another study, Barta et al. [26] studied the process economics of combined ethanol, biogas, heat and electricity production from industrial hemp. One of the results in the study is that it is important to maximise the recovery of potential energy carriers in the raw material, since feedstock is the largest cost contributor in the process. Similar studies have been presented by Dias et al. [27] on improvement of distillation, cogeneration systems and heat integration based on sugar cane bio-ethanol production, while Joelsson et al. [28] and Börjesson et al. [29] studied potential biorefineries, using agricultural and forestry residues, in terms of techno-economic factors, system integration, LCA and feedstock supply. The study aimed at finding suitable locations for a full-scale refinery in Sweden where various factors were evaluated, such as integration with pulp mills, export of heat to district heating systems, etc., Budzinski et al. [30] utilised a hybrid LCA multi-objective optimisation model to evaluate two constraints. The first was based on maximising profit, while the second was based on minimising global impact on climate change. In the study, ethanol production was found to be more cost-effective than ethylene production; however, in terms of reducing climate impacts ethylene production was the better alternative. It must be stressed that techno-economic evaluations, as well as LCA cannot be performed without reliable experimental data. In addition, a rather detailed process design together with information on supply, logistics, economic details, etc., must be available.

## Biomass

“Biomass” is a description for a very heterogeneous group of materials, which sometimes involves also microorganisms. In this review, the term is used for various plant-based material, such as agricultural, forest and herbaceous matter. Despite great efforts invested worldwide to improve cellulose digestibility, cellulosic biofuels and chemicals have yet to be cost-competitive with their starch-based counterparts. The challenge is primarily due to their high production costs associated with pretreatment and enzymatic hydrolysis (and the raw material itself). The variety of plant species is immense, which has a direct impact on the process concepts for their utilisation. The availability of various types of lignocellulosic materials varies from country to country, and continent to continent. In certain regions, forests are abundant, while agricultural species are more common in other regions. Lignocellulosic materials differ from one species to another. However, the main constituents are basically the same, although the contents of individual carbohydrates, aromatics and other compounds vary: about 50–60% are carbohydrates, i.e. cellulose and hemicelluloses, 20–30% lignin, while the rest consist of extractives, fatty acids, ash, etc. [31]. Cellulose is made up of cellobiose units, while hemicelluloses finds their structure from a mixture of hexose and pentose sugars, in combination with various organic acids [32]. Hemicelluloses are more hydrophilic than cellulose and easier to hydrolyse. Lignin is the major non-carbohydrate constituent in lignocellulosic materials comprising a very complex structure of aromatic compounds. It is associated with both cellulose and hemicelluloses and is an important reason for the high strength of many lignocellulosic materials. In general, agricultural crops and hardwood contain more pentose sugars than does softwood [33]. The predominant sugar in most softwood species is mannose, while in hardwood and agricultural species, pentose sugars are in majority [33].

One of the major challenges that the biorefinery concept faces to become successful is to find suitable raw materials. It is likely that second-grade or waste materials will be the main raw material supply in a biorefinery. This includes straw, bagasse, tree roots, branches, forest thinnings, etc. However, a large part of the published research that deals with woody materials are often based on wood chips of high quality. This is in direct competition with, e.g. the interests of pulp producers. On the other hand, the residues from agricultural operations are in many cases available for conversion to other valuable products. However, to maintain soil quality, such as the level of nutrients, not all straw can be removed from the field; an alternative is to return processed residues to the field. Other types of waste materials include municipal solid waste, waste textile, waste-construction materials, etc. Thus, it calls for very robust and versatile production methods to be able to handle raw materials of different origin. It is not likely that a biorefinery will be capable of processing all sorts of lignocellulosic materials.

### The purpose of biomass pretreatment

The purpose of the biomass pretreatment step has somewhat shifted during the last decade(s); previously, the main interest was to use lignocellulosic materials for mainly bioethanol production. The interest in the other main compounds, lignin and hemicelluloses, was limited. Today, it is of great importance to find ways to maximise the overall yield of the valuable compounds that make up lignocellulosic materials. Pretreatment methods that enable efficient recovery of carbohydrates as well as lignin are desired; however, this all depends on the situation and the final product. The energy requirements in the production process must be met under any circumstance, either by internal or external integration of high-energy streams, such as in a mill producing pulp, where the excess lignin is the main process-energy supplier. It has been estimated that—in an optimised mill—between 20 and 30% of the lignin is available for other purposes than for internal energy requirements [34].

Pretreatment is a step that is included as one of the first steps in the process, to alleviate access to the raw material. It is difficult to define “the best” pretreatment for all situations and raw materials; however, it is vital that some important features of the pretreatment method are fulfilled, such as a high recovery of the individual polymers and other compounds in the lignocellulosic material. In addition, the formation of toxic or inhibiting compounds must be low to decrease the risk for negative effects in, e.g. the enzymatic hydrolysis and fermentation steps, if they are part of the process. It is well known that too severe conditions during pretreatment will cause greater degradation of hemicellulosic sugars, which can cause formation of highly toxic compounds, such as furfural, HMF and organic acids. In addition, a plethora of other compounds may be generated; however, furfural and HMF are often used as proxies for the general content of inhibitory compounds [35]. Preferably, the energy requirement must be as low as possible. It is also an advantage if energy integration between the pretreatment step and other parts of the production facility can be implemented, such as utilisation of low-grade steam for distillation purposes. It has also been established that the economic success of a biorefinery is heavily dependent on the solid content of the pretreated materials. If too dilute solutions are produced the energy costs for purification may be prohibitively high, which can cause an otherwise well-functioning pretreatment method to be discarded [36].

The classical way to discuss pretreatment methods has been to divide them into chemical, physical and mixtures thereof, denoted physico-chemical. This is a somewhat arbitrary classification, which largely becomes more and more out-dated since the main aim of a successful pretreatment is now not only to achieve a cellulose fraction that can be easily hydrolysed at a high yield, but also to take care of other constituents. Various forms of “pretreatment” methods have been used for a long period of time. Already in the 1940s, the Scholler process was a means to hydrolyse wood for production of ethanol for fuel and chemicals. However, the Scholler process is strictly speaking not a pretreatment method, but rather an acid hydrolysis using 0.5% H_2_SO_4_ at around 130° for 45 min to hydrolyse cellulose [37]. The definition of the unit operation pretreatment is rather vague. When does acid-catalysed steam pretreatment of a lignocellulosic material become acid hydrolysis instead? Can soda cooking of spruce be considered as pretreatment? In general, a pretreatment step is combined with some kind of another treatment, such as enzymatic hydrolysis; however, several of the rather new methods are largely efficient fractionation methods, which makes it difficult to put a label that clearly defines “pretreatment”.

The research area concerning biomass pretreatment is very active. A database search using the keywords “biomass AND pretreatment” in Scopus for the period 1973–2019 results in about 9600 publications, with a suddenly increasing number of publications starting around 2010. At this time, the number went from about 300 to presently more than 1000 publications per year having the aforementioned keywords. One of the reasons for this may be a more efficient classification system, where the authors’ select proper keywords, but it also shows the continuous high interest for fractionation of various lignocellulosic materials. It is thus an overwhelming task to cover every aspect of biomass pretreatment in all its forms. Therefore, this review is a selection of currently “hot” methods, as well as a historical perspective of methods that still attracts much attention.

### How can different pretreatment methods be assessed?

One of the challenges in selecting a pretreatment method is to evaluate the effect of different methods. It is in general not enough to calculate recovery and yields of the main compounds in the lignocellulosic raw material. These figures are very important, especially when considering the economics of a process. However, they do not fully tell if a pretreatment method is suitable for a certain post-processing step. For instance, if the purpose of pretreatment is to produce ethanol, then several indicators can be used to assess the pretreated material. These indicators include estimation of the levels of inhibitor or toxic compounds, such as aldehydes, organic acid, ketones, phenolic compounds, etc., which may have a detrimental effect on enzymatic hydrolysis and fermentation. A seemingly successful pretreatment, in terms of hydrolysis efficacy and yield, may be far too toxic for a fermenting organism to cope with, which can result in a stuck fermentation, such as after a too severe acid-catalysed pretreatment [38]. On the other hand, if the purpose is to produce, e.g. polymers, gels or binders, other evaluation criteria must be employed. These can include mechanical strength and swelling properties of the polymers [39, 40]. Regarding lignin, its reactivity can be an appropriate indicator [41].

Since several platform chemicals have their origin in monomeric sugars, (e.g. glucose), enzymatic hydrolysis of the pretreated material is probably the most important indicator. This can be a very time-consuming procedure, since enzymatic hydrolysis requires a rather long residence time, often between 24 and 96 h. An approach to reduce the total time and the need for many reactors is described by Studer et al., who developed a high-throughput reaction system [42, 43], where a 96 well-plate unit was employed for both pretreatment and enzymatic hydrolysis. In the system, a 1% solids suspension of a genotype of *Populus trichocarpa* was tested with an excess of cellulolytic enzymes to speed up the assessment procedure. Costa et al. [44, 45] report in studies concerning sugar cane hybrids, a correlation between microscopic characteristics and chemical composition regarding recalcitrance of the sugar cane. Rapid, simple, and efficient screening protocols will be more and more important when selection of modified plants for biorefinery purposes is based on certain properties, such as easily accessible carbohydrates for further processing. In addition, the rapidly growing interest for lignin also shows the requirement for procedures to validate the quality and reactivity of the fractionated lignin.

It has to be stressed that although rapid screening methods are of great value, the final testing must be carried out at higher solid content than 1–2%. The impact on the overall process costs is very much influenced by the achieved concentrations of sugar or other products. A low concentration adds to the separation and recovery costs such that they may become far too high. It is especially important to perform high-solids enzymatic hydrolysis since the conversion of the lignocellulosic materials may become much smaller than expected from low-solids experiments [46].

### Modification of lignocellulosic species

Modification of the plant materials on a genetic level is starting to increase in importance. An example is a study by Holwerda et al. [47], who compared transgenic switchgrass plant lines with their non-transgenic controls. The results show that it is possible to achieve high total-carbohydrate solubilisation, which is dependent on a number of factors, such as feedstock modification, feedstock choice, biocatalyst choice and augmentation of biological attack (i.e. pretreatment). The latter was performed using either ball milling or CELF (Cosolvent-Enhanced Lignocellulosic Fractionation, a method that involves treatment with aqueous tetrahydrofuran and dilute acid at elevated temperatures). Shen et al. [48] modified genetically switchgrass to reduce lignin content, resulting in less formation of phenolic degradation products, while maintaining carbohydrate levels. In another study, Mansfield et al. [12] subjected poplar trees with altered lignin content to steam explosion or organosolv pretreatment (using aqueous ethanol and sulphuric acid as catalyst). Their conclusion was that the low-lignin poplar trees showed a 15% improvement in the conversion efficiency resulting in near-complete hydrolysis of the poplar carbohydrates. It has to be mentioned that enzymatic hydrolysis was performed at 2% dry-matter content, which as discussed earlier, is a non-realistic dry-matter concentration in full-scale operation. It is, however, a common method to assess various pretreatment methods. Wilkerson et al. [49] carried out a study on poplar where they introduced ester linkages in the lignin structure to improve cell wall digestibility after a mild alkaline pretreatment. The concept of tailoring plants to use cell-wall biosynthesis to modify the naturally occurring variety may be an option to grow plants that are more easily deconstructed for biorefinery purposes, according to Wilkerson et al. Another study on genetically modified poplar was carried out by Biswal et al., who showed that it was possible to reduce recalcitrance, have more easily extractable cell walls as well as a more rapid growth in the selected poplar species (*Populus deltoides*) [50]. In addition, the xylan and the pectin contents were reduced.

### Pretreatment and fractionation of lignocellulosic materials

The traditional manner of discussing pretreatment methods is often based on a rather loose classification. In general, the pretreatment methods are regarded as belonging to one of the following categories [51–53]: physical (e.g. milling, grinding, irradiation, sonication); chemical (e.g. alkali, acid, oxidising agents, organic solvents, ionic liquids and deep-eutectic solvents); physico-chemical methods (e.g. steam pretreatment w/wo catalyst, wet-oxidation and hydrothermolysis), or biological methods. In reality, many variations may be made up of combinations of two or more methods.

Another classification can be made according to the pH of the selected pretreatment method, if pretreatment is performed under acidic, neutral or alkaline conditions. This classification is, of course, not valid for methods where an organic solvent, an ionic liquid and similar dissolving agents are utilised for pretreatment (unless a catalyst is added that affects the pH). The pH has a very large influence on the outcome of a pretreatment process. These effects can be described roughly according to the following:

- Low pH, where the desired result is hydrolysis of the hemicelluloses part to monomeric sugars, without formation of inhibiting or toxic compounds, while at the same time keeping the cellulose polymer intact.
- Neutral or close to neutral pH, which result in partial hydrolysis of the hemicelluloses caused by organic acids in the lignocellulosic materials (autohydrolysis). Since the conditions are not severe enough, most of the hemicelluloses will stay in oligomeric or polymeric form. Lignocellulosic feedstocks that are low in organic acids, such as softwood, will not result in high solubilisation of hemicelluloses.
- High pH, which can result in dissolution of the lignin fraction (such as in pulping of wood, or when organic solvents are employed) while some or most of the hemicelluloses still is found in its solid state.

**Fig. 2:**
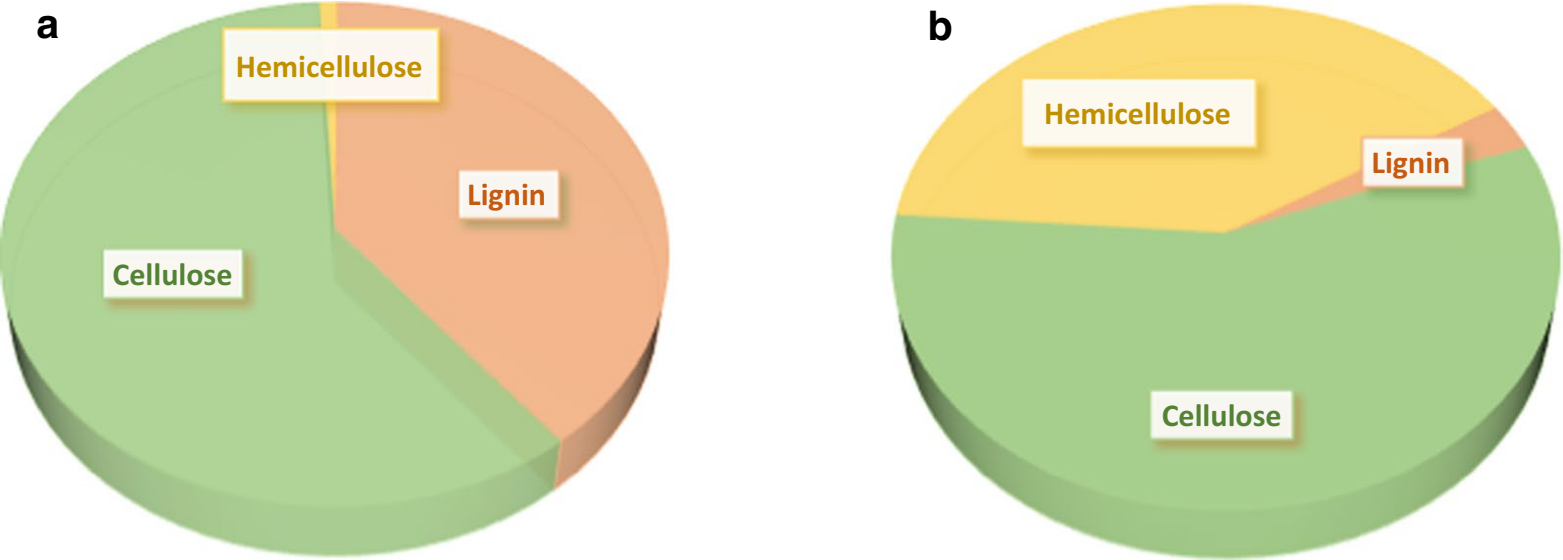
The effect of pH on pretreatment of lignocellulosic materials: **a** low pH; **b** high pH

Acidic conditions result in hemicellulose hydrolysis, which also commonly results in formation of degradation products, such as furfural, HMF, levulinic acid, etc. Carvalheiro et al. [54] have summarised several pretreatment methods where pH is the main cause for the outcome of the procedure. It is obvious how the distribution of the main polymers in a lignocellulosic material changes as the pH moves from low to high values. At low pH, almost all hemicelluloses are removed from the solid material; in contrast, at high pH, lignin is dissolved and cellulose and hemicelluloses constitute the solid fraction (Fig. 2).

The classification can also be based on the expected fractionation result. Many pretreatment methods today are based on solvents (or regarding their action, solvent-like chemicals), yielding results, which to some extent resembles the outcome from high-pH pretreatment methods, i.e. a solid phase containing relatively higher parts of hemicelluloses and cellulose, while most of the lignin is dissolved. It is also in this research area where a multitude of combinations of chemicals is under intense scrutiny, both regarding their suitability for pretreatment and fractionation of lignocellulosic materials, but also regarding concerns for health and process safety. However, the somewhat older established processes are also under constant development, often in combination with studies on lignocellulosic waste materials from all kinds of agricultural, woody or herbaceous plants. Much of these residues are currently of no or low value, but still represent a considerable amount of biomass that could be used for production of value-added chemicals and fuels without too complicated process designs. A comment about their low value is in place here: as soon as a waste can be utilised, the material cost is likely to increase, which is one of the reasons why it is difficult to make economic estimations for a full-scale production facility.

In Table 2, a selection of commonly utilised pretreatment methods is summarised. As already pointed out, the vast number of pretreatment-related publications makes it difficult to discuss in detail every pretreatment method. This review summarises frequently applied pretreatment methods, which can be applied in a biorefinery facility.

**Table 2:** A selection of commonly utilised pretreatment methods

Method	Active agent	Mode of action
Dilute-acid pretreatment	H_2_SO_4_, H_3_PO_4_ and other strong acids	Hydrolysis of hemicelluloses
Alkali pretreatment	NaOH, lime, Na_2_CO_3_ and similar alkaline compounds	Extraction of lignin
Steam pretreatment/steam explosion	High-temperature steam; catalyst may be added	Hydrolysis of hemicelluloses, fibre separation
Ionic liquids	Large organic cation and a small inorganic anion	Fractionation of polymers
Deep-eutectic solvents	Mixtures of Lewis and Brønsted acids and bases	Fractionation of polymers
Organosolv	Organic solvents, e.g. ethanol, butanol. Catalyst can be added	Extraction of lignin
Milling/grinding	Particle size reduction	Surface increase and improved access
Biological treatment	Degradation of the material	Brown-rot degrades hemicelluloses and cellulose White-rot break down lignin Soft-rot breaks down cellulose

## Pretreatment methods

### Acid and alkaline methods

In this section, *acid and alkaline methods* based on the hydrolytic action at low or high pH are discussed. At low pH, the hemicelluloses fraction hydrolyses, while the cellulose and lignin fractions in general are less affected. At higher pH, it is instead lignin that is solubilised, which forms the basis for many pulping processes for production of high-quality journal paper. Depending on the severity of the pretreatment, the extent to which the solid material dissolves can vary to a high degree. If a high acid concentration is employed at high temperatures, not only hemicelluloses but also cellulose converts to oligo- or mono-saccharides. Under even more severe acidic conditions the carbohydrates degrade to other compounds, e.g. furfural, HMF, levulinic acid, etc. Typically, temperatures ranging from 140 to 200 °C and a residence time of minutes to hours are common. Alkaline conditions often have the greatest effect on agricultural residues or herbaceous crops, as these materials in general contains less lignin [55, 56]. An excellent techno-economic analysis of six pretreatment technologies (ammonia fibre expansion, dilute acid, lime, liquid hot water, soaking in aqueous ammonia and SO_2_-impregnated STEX) was published 2011, in the CAFI project (Consortium for Applied Fundamentals and Innovation) [22], where switchgrass was used for evaluation of the processes. Interestingly, the result shows that the differences between the technologies were limited. However, switchgrass is not very recalcitrant in contrast to softwood, which may have exhibited larger variation of the estimated costs.

Dahunsi [57] studied the effect of acid or alkaline hydrogen peroxide pretreatment on pineapple peel prior to anaerobic digestion. In the study, based on response surface methodology, they varied the temperature between 75 and 115 °C, the residence time between 6 and 46 min, the H_2_SO_4_ concentration was 0–2% (v/v). A similar procedure was employed using alkaline H_2_O_2_. The main results from the study were that alkali-enhanced pretreatment yielded a lignin reduction and a biogas production, which was 67% higher than the corresponding acid treatment. Another observation was that the residence time in the biogas reactor was shorter for the alkaline-treated pineapple peel. In another study by Harun and Goek [58], NaOH was used to carry out pretreatment on rice straw. The straw was pretreated at 55 °C for 1 and 3 h, respectively. A concentration of 2–12% (w/v) of NaOH and a ratio of rice straw to NaOH solution of 1:20 (w/v) was applied to all samples. One of the results was that an increase of the glucan content by 85.6% (relative to untreated straw) was achieved using 12% NaOH for 1 h. This condition also resulted in the highest delignification of the straw, 79.6%. On the other hand, straw treated for 3 h with 2% NaOH was found to give the highest total carbohydrate content, 79.2%.

An example of the current biorefinery trend, where valuable lignocellulosic constituents are utilised for production of value-added compounds, is the integrated process for coproduction of fermentable sugars and lignin from poplar pellets that was evaluated by Chu et al. [59]. The process was based on alkali-sulphite pretreatment for production of lignin adsorbents and sugars. In the process, Na_2_SO_3_ was used to soak poplar wood pellets prior to a two-stage cook, the first at 130 °C for 30 min, while the second was carried out at 210° for 5 min. After pretreatment the liquor and the solid residue was separated. After washing, enzymatic hydrolysis on the solid residue was performed. The liquid fraction was processed further using nitric acid to yield an adsorbent product. Over 75% of the original carbohydrate content was released and potentially could be used for fermentation. The adsorbent was tested using lead ions, which resulted in adsorption capacities of 156 and 68 mg g^−1^, for lignin from pre-hydrolysate and enzymatic hydrolysis residue, respectively. The probable reason for the heavy-metal ion adsorption capacity was suggested to be incorporation of sulphur-containing groups in the lignocellulosic material.

Dilute-acid pretreatment of rice straw in pilot-scale was carried out by Kapoor et al. [60]. A continuous pilot-scale reactor was utilised to pretreat rice straw at a feeding rate of 10 kg h^−1^ at temperatures ranging from 150 to 166 °C with a fixed residence time of 10 min. All experiments were conducted for 4 h to secure steady-state conditions. The pH of the slurry was then adjusted to 5.2, after which enzymatic hydrolysis was carried out. Pretreatment using 0.35 wt% acid, 162 °C and 0.65 wt% acid at 152 °C yielded almost the same result in terms of sugar recovery. Overall, about 65% of the potentially available carbohydrates could be recovered. It was also pointed out that a higher recovery can be achieved by adding more enzymes; however, there is as trade-off between the efficacy of enzymatic hydrolysis and enzyme dosage since enzymes are expensive.

The SPORL method (sulphite pretreatment to overcome recalcitrance of lignocellulosic) is a technology utilising sulphite in the pretreatment step. This pretreatment technology allows lignocellulosic materials to react with a solution of a sulphite salt (with e.g. Na, Mg, or Ca) at a temperature of 160–180 °C, and a pH of 2–4 for about 30 min [61]. After the sulphite cooking, a disk milling operation takes place to generate a fibrous material for subsequent saccharification (and fermentation). When spruce wood was subjected to SPORL treatment, the overall yield after the saccharification step was found to be 87.9% for glucose and xylose. During the process, hemicelluloses were dissolved, and about 32% of the lignin fraction was dissolved and sulphonated. The SPORL process was compared with a dilute-acid pretreatment performed at the same temperature and residence time, which resulted in about 57% recovery of the sugars.

Another technology that has been studied in pilot-scale is the ammonia fibre-expansion method (AFEX). Sarks et al. [62] investigated in two 450 L packed-bed reactors pretreatment of corn stover followed by enzymatic hydrolysis and fermentation. The water content in the corn stover was adjusted to 25% before being heated to 80°. Then, biomass was treated with gaseous ammonia at a ratio of ammonia to biomass of 1:1 for 30 min. One of the interesting results is the increase in yield relative to that of laboratory-scale experiments (10 L). The glucose and the xylose release after 48H increased by 19 and 15%, respectively, in the pilot-scale experiments. In addition, ethanol production showed a 15% increase compared to laboratory-scale trials. The reasons for the higher conversion is not fully clear. However, improvements were seen already earlier when the transition from a stirred batch reactor to the laboratory-scale equipment was made. The improved conversion has a direct effect on the minimum ethanol-selling price, which was estimated to be about 19% lower.

Biomass particle size is reported to have an effect on the distribution of the constituents of the biomass. In a study on switchgrass, Bridgeman et al. [63] found that smaller particles have a significantly larger content of inorganic matter than larger particles. In contrast, the larger particles had a relatively higher content of carbohydrates, but were lower in nitrogen content. The influence of particle size on AFEX-pretreated rice straw was studied by Harun et al. [64] who pretreated milled (2 and 5 mm) and cut (2 and 5 cm) particles. In addition, two levels of severity were employed for each particle size. The less severe experiments were performed at 100 °C for 30 min at a straw: ammonia ratio of 2:, while another set of experiments were conducted using more higher temperature (140 °C), longer residence time (50 min), and a ratio of 1:1 of ammonia to straw. Enzymatic hydrolysis of the AFEX-pretreated fractions showed an impact of particle size, in that larger particles resulted in lower sugar conversion for the less severe conditions. In contrast, the more severe conditions yielded a higher conversion with increasing particle size. The largest, cut particles significantly demonstrated a higher conversion than did smaller particles at the highest severity.

The AFEX technology has also been employed for production of livestock feed. The technology was used to upgrade wheat straw in an attempt to reach fodder-level quality. The true IVOMD (in vitro organic matter digestibility), which is a measure for fodder quality, improved on average by 193 g kg^−1^ to reach an average of 844 g kg^−1^, which can make a large difference in many countries. The authors point out the importance of testing the pretreated straw regarding the health effects on the animals and, in addition, if the milk and meat are safe for human consumption. Bals et al. [65] carried out a study on the concentration of acetamide (an ammoniation by-product) in AFEX-treated crop residues and in milk and meat from cattle and buffalo that had been fed with the material. When the AFEX-treated material was introduced, the levels of acetamide in the blood increased. It is possible that the levels will decrease over time due to an adaptation by the animal; however, continued research is necessary to assess the risk, if any, for ruminants and humans.

### Steam explosion (pretreatment) and extrusion methods

Steam explosion (or steam pretreatment) is one of the most widely investigated methods and has been so for a long time. In Table 3, a selection of results from steam pretreatment using typically investigated materials is presented. STEX has been used for many purposes on a large range of lignocellulosic materials, and in combination with other pretreatment procedures. This includes studies on liquid and gaseous fuels production, e.g. ethanol and butanol, or biomethane through anaerobic digestion, respectively. The variation in lignocellulosic materials treated by STEX is very large. They range from food-industry waste to forest and agricultural residues, but also include wood chips from high-quality wood. It is obvious that STEX still is of high interest for biorefinery applications, based on the large number of publications, both past and present.

**Table 3:** Selected steam pretreatment results using various lignocellulosic materials

Biomass	Catalyst/procedure	*T*, °C	*t*, min	Main product(s)	Yield(s)	Refs.
Alpine hay	No catalyst	160–220	5–15	Glucose, biogas	Glucose: 90% Biogas: 469 L_N_	[67]
Poplar wood	Mechanical refining + STEX Neutral sulphonation	210	5	Carbohydrates Sulphonated lignin	Carbohydrates: 81%	[68]
Hybrid poplar	4.5% (w/w) SO_2_	195	4.5	Ethanol prod. for evaluation of modified lignin content	15% improvement in ethanol for low-lignin content breeds	[12]
Sugar cane bagasse	Pre-soaking in reverse osmosis water	185–215	10–15	16 g/100 g DM (whole slurry)	Max combined carbohydrate yield: 65%	[69]
Corn cob	0.5% H_2_SO_4_ (12 h)	180	10	Lignin	57.3% (purity 99%)	[70]
Wheat straw	1% acetic acid	190	10	Ethanol	0.32 g/g (of glucose and xylose)	[71]
Agave bagasse	No catalyst	142–179	2.8–22	Biogas	BM: 0.290 L_N_ g_COD_^−1^	[72]
Rice straw	Moisture content 0–70%	160–205	1–10	Biogas	Increase in production rates up 2.4 time untreated straw	[73]
Switchgrass	Hydrated with water to 30% DM	170–200	5–15	Glucose	After EH: 88.3%	[74]
Sesame seed	Soaked in water (12 h)	212 (2 MPa) 180 (1 MPa)	10 s 30 s	Lipid extraction	45% (treated) 38% (untreated)	[75]
Barley straw	No catalyst	180	30	Ethanol	50 g L^−1^ (99% cellulose recovery; 82% hemicelluloses after STEX)	[76]
Wheat straw/corn stover (mixed)	Soaking 0.2% H_2_SO_4_	190	5	Ethanol	> 50 g L^−1^ Overall yield 74–78%	[77]

The term “steam explosion” (STEX) is originally based on the notion that steam, under high pressure inside the cell structure, is rapidly released and expanded when the reactor vessel is de-pressurised. This causes disruption of the cell structure similar to that of an explosive action. The term “steam explosion” is somewhat misleading since an explosion in the physical sense does not take place. However, since there is a loud bang when the material is suddenly de-pressurised, and the material can be brought open to show individual fibres, the term is still widely used. The background is the Madison process for production of fibreboard, which was described already in 1930 [66]. In the process, wood chips are subjected to steam at around 265 °C for a short time, and then pressed to a fibreboard material.

It is not completely clear if the explosion itself is necessary to yield an easily hydrolysable material. Brownell et al. [78] investigated the mechanical effect of the pressure drop by comparing two sets of experiment: one with a rapid pressure release, and another where 80% of the steam was vented off prior to complete de-pressurisation. The result was that there was no significant difference in the yields of glucose whether the release was from full steam pressure, or from a reduced pressure.

Muzamal et al. [79] evaluated three effects of STEX pretreatment on Norway spruce wood in specially designed STEX experiments at temperatures ranging from 165 to 195 °C:

- The influence of STEX time and pressure,
- The effect of pressure-release rate (slow or rapid),
- The effect caused by shearing between the chips, and the material impact with the vessel walls.

Their study showed that STEX pretreatment clearly increases the porosity of the wood fibres. The explosion step alone does not disintegrate the chips. The impact of highly softened wood chips grinding other chips, and effect from the impact against the vessel walls were more important in this regard. STEX enlarges pores in wood through chemical changes during steam treatment, by opening the wood structure with the expansion of vapour inside the tracheid. In summary, all three steps were considered to have a synergistic effect that increases the effectiveness of STEX pretreatment.

Similar studies were performed by Jedvert et al. [80] who utilised mild STEX for production of pulp from Norway spruce (*Picea abies*). The spruce wood was subjected to temperatures between 115 and 160 °C for 10 min. The study aimed to study the effect of the process conditions on hydrolysis, for kraft cooking of spruce chips, and for alkali extraction experiments. Their conclusion was that mild STEX makes the wood structure more accessible for enzymes and for pulping. Thus, the implementation of mild STEX prior to further treatment can be beneficial as a first step in biorefinery concepts, where further processing by enzymes are planned. In addition, traditional pulping can be improved by such a treatment, since the distribution profile of cooking chemicals inside the chips appear to be more even. In the study, one of the conclusions was that at lower temperatures, the mechanical effect of STEX was of importance.

The size of the chips or straw pieces is likely to have an effect on the overall enzymatic sugar release. Standard pulp chips are rather well defined, about 30 to 50 mm in the fibre direction and 5–10 mm in the cross-fibre direction. Further size reduction can consume large amounts of energy; thus, it is preferable to avoid additional size-reducing steps. DeMartini et al. [81] steam-pretreated aspen (*Populus tremuloides*) using a standardised wood-chip size at 180 °C for 4–18 min. The resulting pretreated materials were subjected to enzymatic hydrolysis for a residence time ranging from 24 to 168 h. They experiments were evaluated by MRI, SEM and staining techniques. The study shows that it is possible to have a uniform pretreatment also for wood chips of industrial size. Their conclusion was that the rapid decompression achieved by steam explosion might alter accessibility at lower temperature conditions.

Vidal et al. [82] reviewed the effect of particle size for various pretreatment methods. Particle size may have a direct effect on enzymatic hydrolysis. The particle size is, however, less important than the removal of, e.g. hemicelluloses and lignin. By employing physical size reduction, conversion yields of about 50% can be achieved. Thermochemical pretreatment methods can handle particle sizes to a varying degree. The maximal size range that a certain pretreatment method can handle is stated to be decreasing as follows: STEX > LHW > dilute acid and alkali. The feedstock also appear to influence the outcome of EH; thus, herbaceous or grassy biomass exhibits a lower maximal size (< 3 mm) than woody biomass. Another aspect is how the lignin structure is affected by the STEX process. Wang et al. [83] used several analytical methods such as SEC, FTIR and NMR(HSQC) to study the effect of STEX on poplar wood, which was treated at 209 °C for 7 min. Samples from native poplar wood, as well as from the STEX-treated solid and liquid fractions were evaluated. The results show that STEX pretreatment reduces the amounts of β-O-4′, β-β, and spirodienone structure, and increases the syringyl/guiacyl ratio from 1.14 (native poplar) to 1.70 (solid) and 1.86 (liquid), respectively. In addition, SEC results showed that de- and re-polymerisation are the main reasons for the increase in lignin average molecular weight in the solid fraction, while it decreases in the liquid fraction. An important conclusion is that although the lignin structure changed, the backbone structure was not modified to a large degree.

Thus, although STEX has been studied for many years, the conclusion from these studies shows that the overall effect of STEX is still not clear. However, the higher the temperature, the more important the chemical effect becomes, i.e. the reactions caused by acids and other degradation reactions inside the lignocellulosic material. The approach to use continuous steam-pretreatment equipment where the lignocellulosic material is transported through the reactor is also a means to increase the reaction rate in comparison with non-stirred batch equipment. In a continuous reactor, the mixing properties are improved largely. Mixing makes it possible to have less severe conditions during the pretreatment step, which is beneficial since non-desired side reactions, such as furfural formation, is promoted by higher temperatures. Cornejo et al. [84] used thermochemical pretreatment (steam pretreatment) equipment to pretreat poplar, wheat straw and pine employing sulphuric acid as a catalyst (1–3% for wheat straw; 0–4¤ for pine and poplar). The selected temperatures were between 164 and 192 °C for poplar and pine chips, and ranging from 173 to 187 °C for wheat straw. The residence times were between 5 and 15 min. The purpose was to solubilise the hemicelluloses fraction for transformation to furfural, and for production of glucose. Very high conversions of cellulose to glucose were achieved after enzymatic hydrolysis: poplar biomass resulted in the highest yield (40 g/100 g DM), while pine only yielded 25 g/100 g DM) over the enzymatic hydrolysis step. The results are in line with previous results where pine has been shown to be difficult to hydrolyse efficiently [85].

Del Carmen Fong Lopez et al. [86] studied a continuous process combining an alkaline pretreatment and neutralisation in a twin-screw extruder. In the study, dehydrated sweet corn co-products (SCC) were exposed to NaOH in concentrations between 3.9 and 8.0% w/w and temperatures ranging from 52 to 168 °C. The accessibility of the solid extrudate was assessed by enzymatic hydrolysis at 5% DM. At optimal conditions, a resulting cellulose conversion of about 70% was achieved. The authors state several phenomena that may be involved in the extrusion process. These include structural biomass modification. In addition, the sugar yield could be improved by a higher temperature, while decreasing the amount of catalyst. To some extent, extrusion of biomass is similar to the continuous steam-pretreatment method discussed earlier. The motion and the mixing of the lignocellulosic materials improves heat and mass transfer in the reactor. However, the extrusion process results in a much more intense abrasion of the material.

Steam explosion has been used not only as a preparative step prior to enzymatic hydrolysis for further processing for the sugar-platform concept, but also for enhancing biogas production [67]. In a study performed by Bauer et al., hay was steam pretreated at temperatures ranging from 160 to 220 °C in intervals of 15 °C. The residence times were 5, 10 or 15 min. Subsequently, the steam-pretreated materials were enzymatically hydrolysed. In comparison with untreated hay, the methane yield increased by about 16%.

### Hydrothermal methods

*Hydrothermolysis* is a method that uses liquid water under pressure at high temperatures. The method is also known as hydrothermal treatment, autohydrolysis, pressure-cooking in water, etc. It has mostly been used in studies regarding hardwood lignocellulosic materials [87–89] and annual plants [90, 91]. The mechanism of hydrothermolysis is believed to be working by auto-ionisation of water, causing acetyl groups in hemicelluloses to form acetic acid. The dissociated acetic acid catalyses, e.g. carbohydrate degradation and dissolution of hemicelluloses. Hydrothermolysis has also been evaluated for extraction of lignin and carbohydrates from pine. Ståhl et al. [31] treated pine chips at temperatures ranging from 200–240 °C having a liquid-to-wood ratio of 40:1. Within 10 min, approximately 35% of the lignin was solubilised during hydrothermolysis at 240 °C. This amount of lignin corresponds to about 97 kg per ton of dry wood. If the reaction was extended, increased condensation of lignin took place. Two fractions of lignin were recovered in the liquid phase, which were found to be reactive; in addition, the recovered lignin was sulphur-free, which can be an advantage for use in other applications. The hemicelluloses fraction was found to be completely liquefied already at 200 °C, where also about 12.5% of the cellulose fraction was dissolved. Ståhl et al. also propose a kinetic model for degradation of xylan, mannan and glucan. The model has a number of constants [18] that were fit to the suggested rate equations. Lignin in the solid residue can be calculated from analytical expressions.

The hydrothermolysis technology has also been used for fractionation of agricultural materials such as rapeseed flour into, e.g. amino acids, carbohydrates, fatty acids and organic acids [92]. The treatment was carried out with subcritical water at temperatures between 180 and 280 °C for residence times up to 60 min. The highest yield of amino acids was obtained at 200 °C and a residence time of 60 min (124 g per kg rapeseed flour). However, carbohydrates were obtained at different conditions: xylose and glucose reached maximum concentrations of 51 g kg^−1^ (220 °C, 20 min) and 51 g kg^−1^ (260°, 10 min), respectively. A similar study was performed on pectin from citrus and apple pectin, with the purpose of producing uronic acids [93]. Uronic acids are valuable chemicals that are used in several application, e.g. in the cosmetic industry. The highest yield was 79.7 g kg^−1^ at a temperature of 150 °C and a residence time of 40 min.

A different application than most other hydrothermolysis investigations was studied by Chu et al. [94]. They explored the direct conversion of a sediment from the conventional kraft-pulping process (black liquor acid sediment) into phenolics. Depolymerisation of black liquor lignin is a means to produce intermediates that can be processed further to yield high-value chemicals. In the study, the effects of temperature and residence time were investigated. The temperature was varied between 260–340 °C, and the maximum residence time was 120 min. The yields of four products—oil phase, aqueous phase, char product and gas phase—were evaluated. Their conclusion was that direct hydrothermal conversion of the black liquor is a promising method. The selectivity for phenolic compounds, especially catechol was high.

### Organic solvents/organosolv/green solvents

Pretreatment aided by an organic solvent is known as *organosolv*. One of the important features of organosolv methods is the ability to cause biomass dissolution. The organosolv pretreatment method employs organic solvents of widely different kinds, such as ethanol [95–97], acetone [97], organic acids (e.g. formic and acetic acid) [96], ethylene glycol [98]. More recently, liquids such as γ-valerolactone [99, 100], methyl isobutyl ketone (MIBK) [101], tetrahydrofuran (THF) mixed with water [102] and 2-methyltetrahydrofuran [103] have been investigated. An excellent review by Zhang et al. [96] on numerous solvents that have been experimentally tested for organosolv, discusses in detail pretreatment using organosolv. In the review, the pros and cons of many solvents are reviewed in terms of physical properties (e.g. boiling points, solvent polarity, their rating as a green solvent, etc.); in addition, a thorough survey of the effect of the organic solvent on saccharification using cellulolytic enzymes is presented. Organosolv does—as does alkali—remove lignin in rather large amounts. Alkali mainly works by disrupting the bonds between lignin and hemicelluloses to cleave off ether and ester bonds between carbohydrate and lignin. It also breaks C–C bonds in lignin by hydrolysis [104]. Organosolv acts by cleaving β-aryl-ether bonds either by acidolysis and (or) homolytic cleavage. In addition, some of the hemicelluloses are dissolved and degraded to smaller compounds [105].

Ethanol–water mixtures are still one of the most common ways to perform organosolv pretreatment. A catalyst is often added, which enhances the effect of the solvent. Bouxin et al. [95] examined the effect of heated acidified aqueous ethanol treatment on Sitka spruce sawdust [95]. Sulphuric acid having concentrations between 0.75 and 1.25% (w/w) was used as catalyst. The range of pretreatment temperatures was 150 °C to 180 °C followed by enzymatic hydrolysis. The solid organosolv residue was subjected to a saccharification step. A decrease in lignin content from 35 to 22% did not significantly have an impact on the enzymatic hydrolysis. The highest saccharification yield was obtained at a residence time of 60 min at a temperature of 180 °C, with an ethanol concentration of 60% and 1% H_2_SO_4_. Under these conditions, a saccharification yield of 86% was reached. A reduction of pentose degradation to furfural was also noticed. A large part of the hemicelluloses sugars was also converted to ethyl glycosides, which is a valuable product as an intermediate in the sustainable production of value-added chemicals. Matsakas et al. [106] used a modified steam-explosion reactor for operation with ethanol instead of steam. They pretreated spruce at a temperature of 200 °C, varying the residence time between 15 and 60 min, the ethanol content between 52 and 65% (v/v), and addition of sulphuric acid (0–1% w/w). The pretreated solid material was processed using high-gravity SSF at 22% solid content. The highest resulting ethanol concentration was 61.7 g L^−1^, with a total yield of 68.6% of the theoretical maximum. This is in the same range as reported by Hoyer [107] who reached 65 g L^−1^ and a yield of 72.1% employing STEX.

Recovery of the solvent is of high importance to enable a cost-efficient process, where make-up chemicals should be kept at a minimum. Lê et al. [108] processed *Eucalyptus globulus* wood chips using GVL/water fractionation with different liquid-to-solids ratios ranging from 2 L kg^−1^ to 10 L kg^−1^ at a reaction temperature of 180 °C and a residence time of 150 min. The GVL content in the liquor was 50%. Lignin was recovered from the spent cooking liquor by the addition of water, and the precipitated lignin could be collected by spinning in a centrifuge. The recovery of GVL was performed utilising several separation methods, e.g. distillation at reduced pressure, lignin precipitation by water addition, and liquid CO_2_ extraction. The combination of precipitation and distillation at reduced pressure resulted in 90% recovery of GVL and the formation of a sticky residue, a mixture of lignin and GVL. Distillation at reduced pressure is a rather complex unit operation, which increases the risk for process stability should leaks occur in the distillation unit. However, it is frequently used in the petroleum refineries for the heavy oil fractions. Using liquid CO_2_, 87% of the GVL could be recovered in the extract, and the rest was found in the raffinate. Six recovery schemes were proposed of which two were considered to become feasible given further optimisation. It was also pointed out that the process for water recycling of the washing water must be investigated.

GVL was utilised in a study by Alonso et al. [99]. The solvent has shown high applicability for fractionation of lignocellulosic biomass. By adding an acid to serve as a catalyst, a mixture of GVL, water and acid can dissolve the hemicelluloses and the lignin parts, resulting in a high-purity cellulose residue. The cellulose can be processed further to produce dissolving pulp for textile-fibre production. A potential advantage of GVL is the manufacturing process of the compound itself. GVL is produced from levulinic acid, which is a degradation product from hexoses; thus, through hydrolysis of part of the available cellulose into glucose, hydroxymethylfurfural can be dehydrated and yield formic acid and levulinic acid, which is the precursor for GVL [109]. From a safety perspective, GVL has physical properties that makes it suitable for storage and transportation, e.g. a low vapour pressure (3.5 kPa at 80°) [110]. The solubility of lignin increases with increasing GVL concentration in an aqueous mixture to an estimated optimum of 92–96 wt% GVL. If water is added lignin precipitates, which opens a way for separation and recovery of both lignin and GVL [111].

A combination of two technologies, organosolv and dilute-acid pretreatment was performed by Chin et al. [98] who used ethylene glycol as a first step in pretreating empty fruit bunch at 85° for 45 min with 50% ethylene glycol in the presence of 3% NaOH. The resulting solid fraction was then subjected to a two-stage dilute-acid hydrolysis, where stage 1 was performed with 36–90 wt% H_2_SO_4_ at 80 °C for 45 min. Stage 2 employed dilute acid (3–15%) at a temperature of 100 °C for 60 min. The highest release of sugars was about 90%, while the formation of degradation products was rather low. The best conditions was found to be an acid concentration of 45% in stage 1 at 65 °C for 30 min, followed by a second step using 12% acid at 100 °C for 120 min.

A selection of various biomass materials, which have been pretreated utilising different organosolv-like pretreatment methods, is presented in Table 4. The number of organic solvents, which have been utilised in the organosolv process, is very large; therefore, only some of the typical solvents, as well as some more recently investigated solvents are included.

**Table 4:** A selection of organosolv methods utilised for pretreatment of lignocellulosic materials

Biomass	Solvent	Glucan recovery (%)	Xylan removal (%)	Lignin removal (%)	Comment	Refs.
Hybrid *Pennisetum*	Water Acetone Ethanol THFA GVL	91.1 90.4 93.4 92.3 90.9	36.8 47.8 53.5 46.8 43.4	11.6 32.8 37.6 46.8 50.3		[97]
Coir (coconut fibre)	1,4-Butanediol/acidic IL	87.5–90	77–93	75–88	Combination of organosolv and ionic liquids	[112]
Eucalyptus 1. Bark 2. Wood	Ethanol Oxalic acid Water				1. Glucan content (solids): 75.6% 2. 50%	[113]
1. SCB 2. Tall fescue 3. Sugar beet 4. Eucalyptus 5. Beech 6. Japanese cedar	Butanol	81 80 65 80 81 79		87 87 85 80 72 12	Glucose yield (%) after EH: 1. 77 2. 74 3. 69 4. 65 5. 65 6. 1	[114]
Eucalyptus Bark	Ethanol	74–93	15–70	25–52		[115]
Rice husks	Ethanol	88–90	86.8	77.5		[116]
Empty fruit bunch (palm tree)	Ethylene glycol (3% NaOH)	90.6	10–54	67.2	Starting material was decomposed fruit bunch	[117]
Corn stover	Methanol/NaOH	97.5	16.5	37.3	Enzyme hydrolysis: Glucan: 97.2% Xylan: 80.3%	[118]
Bamboo	Formic acid	98	22	83		[119]
SCB	Autohydrolysis + glycerol	80–90	55–68	48–84		[120]

### Ionic liquids

Efficient pretreatment and fractionation methods for production of value-added products heavily relies on the availability of efficient and not too expensive processes. It is also imperative that the products can be recovered at a high yield using common separation and recovery equipment. The potential candidates for commercialisation must be safe from health and environmental perspectives. A particular interesting group of chemicals is the *ionic liquids*. Ionic liquids are salts that consist of an inorganic anion and an organic cation [121–123]. The cations are typically composed of organic cores, e.g. imidazolium, phosphonium, pyrrolidinium, cholinium and many more [123]. The combination of a large organic cation, in which the positive part is either sterically hindered or shielded, and a smaller inorganic cation, causes the salts to prefer the liquid state in many systems. ILs are usually divided into two classes, i.e. protic and aprotic ILs [124]. The seed for a virtual explosion in the number of ionic liquids was the discovery of the 1-ethyl-3-methylimidazolium cation [EtMeim] in 1982 [125, 126]. A drawback at the time was the requirement to avoid moisture entering the IL system. The development has been rapid since and more and more combinations of salts are utilised for very different purposes, such as man-made cellulosic fibres [127] and for extraction of lignin from biomass [128]. The combination of cations and anions making up ionic liquids are almost endless [127]. The possibility to make combinations of various cations and anions is an advantage, since the properties of the IL may change depending on the selected combination, which opens up for fine-tuning of the pretreatment process. Table 5 presents a summary of various combinations of ionic liquids. The acronyms are not always the same in different publications. As mentioned earlier, the number of potential ILs are very large; therefore, only some commonly utilised ILs are presented. The ILs have been referred to by a number of notations: Room-temperature ionic liquid, non-aqueous ionic liquid, molten salt, liquid organic salt, and fused salt have all been used to describe salts in the liquid phase [129]. They are non-volatile, non-flammable and have high chemical and thermal stability. Room-temperature ionic liquids (RTILs) are of special interest since they can be used at low temperatures (below 100 °C) [129–131].

**Table 5:** A selection of cations commonly used for ionic liquids

Acronym	Cation	Refs.
[Hmim]	1-Methylimidazolium	[132]
[C_4_-py]	1-Butyl-pyridinium	[133, 134]
[Bmim]	1-Butyl-3-methylimiazolium	[127, 132]
[Emim]	1-Ethyl-3-methylimidazolium	[127, 134]
[Mmim]	1,3-Dimethylimidazolium	[135]
[Pdmim]	1-Propyl-2,3-dimethylimidazolium	[136]
[Hexpy]	1-Hexylpyridinium	[134]
[Bmpyr]	1-Butyl-3-methyl pyridinium	[137]
[Hmpyr]	1-Hexyl-3methyl pyridinium	[137]
[Amim]	1-Allyl-3-methylimdazolium	[127]
[C_*n*_pyr]	1-Alkylpyridinium	[138]
[mDBN]	5-Methyl-1,5-diazabicyclo[4.3.0]-non-5-enium	[139]
[DBNH]	1,5-Diazabicyclo[4.3.0] non-5-enium	[139]

Ionic liquids have a potentially wide field of applications for biomass processing. A number of areas have been identified, such as fractionation, conversion into chemicals, dissolution and hydrolysis. Some hurdles are yet to overcome to make ILs entering in large-scale processes [140, 141]. The separation and recovery of the IL from a mixture of carbohydrates, lignin and proteins can result in challenges for large-scale biorefineries [135, 142]. The individual lignocellulosic components, such as cellulose, can be recovered by addition of an anti-solvent, e.g. water [143, 144], acetone [145] or ethanol [146] by, for example precipitation. The ILs are usually quite costly (*c.f.* DESs in the next section) to produce, and the viscosity is high, which makes pumping and mixing quite energy consuming. Another issue is their sensitivity to moisture and the recovery cost, which can be very high. Zhou et al. have evaluated, in a very comprehensive review, a number of recovery options, such as distillation, extraction, adsorption, membrane separation (including pervaporation and membrane distillation), etc. [147]. Distillation has often been suggested as the main unit operation to be used for recovery of the ILs. However, the energy cost associated with distillation is rather high, which may impair the overall feasibility of a large-scale process [147, 148]. Lynam et al. [146] employed direct-contact membrane distillation (DCMD) at low temperatures and ambient pressure (in contrast to pervaporation methods, where lower pressures are used), which was successfully used to separate water from [C_2_mim][O_2_CH] and [C_2_mim][OAc]. The resulting IL–water mixture must be separated prior to IL recycling because water inhibits IL–biomass pretreatment.

The utilisation of ILs has attracted interest regarding their potential effect on environment and health consequences. Gathergood et al. [149] investigated the biodegradability of commonly used ILs. They designed and evaluated ILs containing ester or amide groups in the alkyl side chain. They found that ILs with an ester in a sidechain were generally liquids at room temperature. The [Bmim]-derived ILs were found to have poor or negligible biodegradability. However, if an ester was incorporated in a side chain, the biodegradability increased significantly. The non-toxic character and their bio-degradability has been studied and several publications provide great amounts of information regarding their bio-degradability, eco-toxicity, as well as cytotoxicity [122, 130, 137, 150, 151]. ILs may have an effect on all levels of life; therefore, ILs have been studied in various life forms, including bacteria, fungi, plants, animals, etc. In addition, since ILs are very water soluble, they may have a large environmental impact should accidents occur [122, 150, 151]. It has been shown that the choice of cation is of great importance. The biodegradability of ILs having the cation cholinium (sometimes classified as belonging to the DES family, which is discussed in the next section) is usually high [122, 152].

ILs have been used to pretreat various lignocellulosic materials. Alayoubi et al. [121] used [Emim][OAc] to pretreat three cellulose-containing materials: cotton, spruce and oak sawdust. In addition, they performed enzymatic hydrolysis of the untreated and pretreated materials. In short, 2% (w/w) substrate was added to 10 ml [Emim][OAc] and incubated the suspension at 45 °C for 40 min. After incubation, the pretreated substrate was precipitated by addition of water (2:1 v/v water-to-IL). The solid fraction was enzymatically hydrolysed and fermented to ethanol. The glucose yields after pretreatment was 70% for cotton, 60% for oak sawdust, while spruce sawdust resulted in a yield of only 50%. The ethanol yields were in all cases around 50%. This has to be considered a low yield if compared with commonly reported ethanol yields from other studies performed at higher temperatures [153, 154]. Although the IL pretreatment is followed by careful rinsing of the solid materials, residual ILs may have a toxic or inhibiting effect on enzymes and/or fermenting organisms [137, 155, 156]. Sitepu et al. [157] scanned one hundred and sixty-eight strains of wild yeast (including the *Saccharomyces* genus) for their tolerance to [Emim][OAc]. Based on growth in media containing [Emim][OAc], tolerance levels between 1 and 5% of the IL were observed for more than 80 strains. This indicates that residual IL in the solid material after pretreatment is not a critical issue from this point of view. In a study by Auxenfans et al. [158] on simultaneous pretreatment and enzymatic saccharification of lignocellulosic and cellulosic substrates concerning spruce, utilisation of [C_2_mim][MeO(H)PO_2_ led to significant glucose yields up to a concentration of 30% IL (v/v) in the saccharification step, while [C_2_mim][OAc] was the better IL for oak sawdust. However, beyond an IL concentration of 10% (v/v) the yields of glucose slowly decreased until the enzymatic hydrolysis stopped at 50% IL or higher. Auxenfans et al. also found that β-glucosidase activity was sensitive to [C_2_mim][OAc] [158].

Lignin is of major interest in current biorefinery-related research for production of for example fuels and chemicals [159]. Therefore, pretreatment methods that can extract lignin from lignocellulosic materials in its native form are of particular interest. However, native lignin differs from extracted lignin using available commercial methods. The solubility of lignin that has been extracted from, e.g. cooking of wood for production of pulp, and used for solubility tests in various ILs may not say anything about the ILs capacity to dissolve lignin from a real biomass substrate [144, 160–162]. Similar studies were performed by Achinivu et al. [163] on three different protic IL cations [Mim], [Pyrr] and [Py] using acetate as anion. They also included xylan and microcellulose in the study. All protic ILs were able to dissolve lignin in large quantities, while cellulose was insoluble. Xylan, on the other hand, has a varying solubility in the tested ILs. However, the results cannot be directly transferred to their ability to extract lignin from a lignocellulosic biomass. The amount of extracted lignin after pretreatment at 90 °C for 24 h employing [Pyrr][OAc] was greater than 70% of the original content in corn stover.

The number of publications that presents results from IL pretreatment of various lignocellulosic materials is rapidly growing. In Table 6, a selection is presented of ILs that have been utilised for fractionation of different types of lignocellulosic materials.

**Table 6:** A selection of fractionation methods for of various lignocellulosic materials utilising ionic liquids

Biomass	IL	*t* (h)	*T* (°C)	Main product(s)	Yield(s)	Refs.
Bleached birch kraft pulp	[Emim][OAc] [Emim][DMP] [Emim][Cl] [mDBN][DMP] [DBN][OAc] [DBN][EtCOOH]	3	60	Pulp	2.37^a^; 2.40^b^ 1.28^a^; 1.44^b^ 1.99^a^; 1.35^b^ 3.56^a^; 2.82^b^ 6.56^a^; 1.18^b^ 6.94^a^; 4.35^b^	[139]
Switchgrass	[C_2_mim][OAc] [FurEt2NH][H_2_PO_4_]^c^ [VanEt2NH][H_2_PO_4_] [*p*-AnisEt2NH][H_2_PO_4_]			Glucose Xylose	90–95 70–75	[164]
Oak sawdust Spruce sawdust Cotton fibre	[C_2_mim][OAc]	40 min	110	Glucose	67–79 66–73	[153]
Southern yellow pine	[C_2_mim][OAc]			Holocellulose/lignin	59/31	[145]
a) Triticale b) Wheat straw c) Flax shives	1. [Emim][OAc] 2. [Bmim][Cl] 3. DMEAF 4. DMEAA 5. DMEAG 6. DMEAS	0.5–24	70–150	Lignin Glucose	For a) & 1: Lignin: 52.7% Glucose: >95	[161]
Pine	1. [HBim][HSO_4_] 2. [TEA][HSO_4_] 3. [DMBA[HSO_4_]	0.5–8	120–170	Lignin Glucose	For 3: Lignin: 70 Glucose: 75%	[165]
Rice straw	1. [C_2_mim][Cl] 2. [C_2_mim][Cl/water] 3. [C_2_mim][Cl/K_2_CO_3_]	1	110	Lignin Glucose	For 3: Lignin: 93.7 Glucose: 92.1	[148]
Cotton-based waste textiles	[Amim][Cl]	0.5–150	90–130	Bacterial cellulose	10.8 g L^−1^ of nano-cellulose fibres	[143]
Wheat straw	[Emim][DEP]	10–120 min	25–150	Reducing sugars (RS)	RS: 54.8 g g DM^−1^ [@130°, 30 min)	[166]

^a^Residual xylan

^b^Dissolved cellulose

^c^The ILs utilised in [164] were synthesised from aromatic aldehydes derived from the major by-products of biofuel production from lignocellulosic materials: furfural, vanillin and *p*-anisaldehyde

### Deep-eutectic solvents

*Deep eutectic solvents* (DESs) are a class of eutectic mixtures of Lewis or Brønsted acids and bases, which can be made up from a variety of anionic and cationic species. It is considered to be a new class of ILs, since they have many properties and characteristics in common [167]. They are generally classified according to four classes, type I, II, II and IV and can be described by a general formula *Cat*^+^*X*^−^*zY*, where *Cat*^+^ can be an ammonium, a phosphonium or a sulphonium cation. *X* is a Lewis base, which can interact with either a Brønsted or a Lewis acid [167], and *z* is the number of *Y* molecules that interacts with the selected anion. DESs can be produced relatively easy by mixing the compounds together at moderate temperatures. They usually have low volatility and high thermal stability [168]. The term deep-eutectic solvent comes from the difference in the expected freezing point if two DES-forming chemicals are mixed; when the two compounds are mixed at a certain ratio, to eventually hit the eutectic point, the freezing point becomes much lower than the corresponding freezing points for the pure components. The DES components are reported to be less expensive to produce than conventional ILs. One estimation states that they are about 20% cheaper than ILs, ranging from $20 kg^−1^ for choline chloride and for urea $35 kg^−1^ [168]. However, Socha et al. suggest a price estimate for the IL [C_2_mim][OAc] of approximately $17–25 kg^−1^ [164]. Thus, the production costs are somewhat uncertain. The potential applications for DESs show that they are very versatile, and they have been reported to be used in very different situations, e.g. in the areas of electrochemistry, pharmaceuticals, fossil fuels, food and feed production and for lignocellulosic biomass fractionation [169].

DESs can only be recognised as green solvents if they can fulfil health and safety regulations, but also sustainability criteria [170] just as is the case for ILs. Most DESs are based on the hydrogen-bond acceptor choline chloride. While choline chloride is a common chemical, frequently used in animal feed, it is believed to be safe from a health perspective. It also supports important biological functions in human beings [171]. In addition, several publications show that cholinium-based liquids can allow protein structures and enzyme functions to be maintained or even enhanced.

DESs have been used for various kinds of biomass fractionation tests. Chen et al. [172] evaluated six ternary DESs for fractionation of switchgrass where the hydrogen-bond acceptor was either choline chloride (ChCl) or guanidine hydrochloride (GH). The hydrogen-bond-donators were chosen to be one of ethylene glycol (EG), propylene glycol (PG), glycerine (GLY) or *p*-toluenesulfonic acid (PTSA). The combination GH-EG-PTSA was the most efficient, resulting in a removal of 79% xylan and 82% lignin in 6 min at 120 °C with 10 wt% solid loading. If the solids loading increased to 35 wt%, a removal of more than 60% in 30 min was possible using GH-EG-PTSA and ChCl-EG-PTSA. The resulting cellulose-rich fraction was subjected to fed-batch hydrolysis to a final solids loading of 20%, which produced 128 g L^−1^ glucose.

The hardwood willow was used in a study by Li et al. [173] using ChCL as HBA and one of lactic acid, glycerol or urea as HBD. The combination of ChCl and lactic acid at a 1:10 molar ratio had the highest lignin-extraction efficacy when treated at 120 °C for 12 h. The lignin yield was 91.8%; if the reaction time was prolonged to 42 h, the increase in lignin extraction was only small reaching 94.8%. They also noticed a small decrease in cellulose content indicating that degradation was initiated. The extracted lignin fraction had a purity of about 95%, which is very high; in addition, the ash content was about 0.5%. FTIR, ^13^C-NMR and ^31^P-NMR showed that syringyl and guaiacyl units were the main constituents of the extracted lignin.

Another lignocellulosic biomass, loblolly pine, was evaluated by Lynam et al. [174]. Loblolly pine (also known as Southern yellow pine) is considered as a very recalcitrant material. Three HBDs (formic acid, lactic acid and acetic acid) and three HBAs (choline chloride, betaine and proline) were utilised in various combinations and ratios. Loblolly pine was added to the DESs at a ratio of 1:10. The fractionation was performed at a temperature of 155 °C for 2 h. The washed cellulose-rich fractions were assessed by enzymatic hydrolysis. An initial evaluation of the lignin-solubilising capacity was performed on alkali lignin, xylan from beech wood and medium fibrous cellulose. The DES consisting of formic acid and choline chloride at a ratio of 2:1 exhibited the highest lignin solubility and the lowest cellulose and xylan solubility. This DES was also selected for fractionation of loblolly pine. The resulting glucose yield after enzymatic hydrolysis for 72 h was about 70% of the cellulose content in the untreated material. The lignin-extraction yield was not explicitly presented.

One of the proposed benefits of utilising DESs is the suggestion that the extracted lignin stays in a more reactive form than, e.g. the lignin from STEX pretreatment. Tan et al. [41] applied acidic DESs on oil-palm empty fruit bunch (EFB) for lignin extraction. Nine organic acids were selected as HBDs while choline chloride was the HBA choice. The molar ratios of the various combinations of the DESs were ranging from 2:1 to 1:15 (HBA: HBD), while the ratio of EFB to DES was 1:10 by weight. The suspensions were allowed to react for 8 h at 120 °C. The results show that a higher lignin yield was achieved for monocarboxylic-based DESs, than for the corresponding di- or tricarboxylic acids. The same relationship was also found by Hou et al. [175] for DES extraction of rice straw. Tan et al. suggests that the increase of possible hydrogen-bonding sites in di- or tri-carboxylic acids may restrict the mobility of solvent molecules, which weakens the solvent–lignin interaction thus decreasing the lignin-extraction efficacy. The DES having the highest lignin-extraction capability was shown to be choline chloride: lactic acid, which at a molar ratio of 1:2 extracted more than 60% of the lignin. This lignin also exhibited a reactivity on par with technical commercial lignin, based on the phenolic hydroxyl content in the extract.

The potential of DESs to be used for fractionation purposes in a biorefinery is likely to increase, as the interest for these types of compounds is becoming larger and larger. In Table 7, a selection of DESs, which have been utilised for fractionation of various biomass materials, is presented. It is obvious that not only woody materials are of interest, but also various kinds of agricultural and fruit waste have been evaluated.

**Table 7:** A selection of DESs utilised for biomass fractionation

Biomass	DES	*t* (h)	*T* (°C)	Main product(s)	Yield(s)	Refs.
Lettuce leaves	ChCl:glycerol	3–16	80–150	Bio-butanol	Glucose: 94.9%; xylose: 75% @150°, 16 h	[176]
Corn stover	1. ChCl:formic acid 2. ChCl:urea 3. ChCl:glycerol 4. ChCl:acetic acid 5. ChCL:oxalic acid 6. ChCl:malonic acid 7. ChCl:citric acid	0.5–3	90–130	Bio-butanol	For 1: Glucose: 99% (17 g L^−1^) Butanol: 5.6 g L^−1^ (0.17 g g^−1^ sugar)	[177]
Willow	1. ChCl:lactic acid 2. ChCl:glycerol 3. ChCl:urea	6–42	90–120	Lignin	For 1: Purity: 94.5% Yield: 91.8%	[173]
Switchgrass	1. GH-PG-PTSA^a^ 2. GH-EG-PTSA^a^ 3. GH-GLY-PTSA^a^ 4. ChCl-PG-PTSA^a^ 5. ChCl-EG-PTSA^a^ 6. ChCl-GLY-PTSA^a^ 7. GH-Eg-PTSA^b^ 8. ChClEG-PTSA^b^ 9. GH-EG-PTSA^c^ 10. ChCl-EG-PTSA^c^	0.1		Fractionation: Cellulose Xylan Lignin	Highest removal for 2: Cellulose: 0.70 Xylan: 79.4 Lignin: 82.1 @6 min, 120 °C	[172]
Corncob	1. ChCl:glycerol 2. ChCl:lactic acid 3. ChCl:glycolic acid 4. ChCl:levulinic acid 5. ChCl:malonic acid 6. CHCl:glutaric acid 7. ChCl:oxalic acid 8. ChCl:ethylene glycol 9. ChCl:glycerol	24	90	Lignin Glucose	For 1: Lignin: 71.3% Glucose: 96.4% (after EH)	[178]
Potato peels	1. ChCl:glycerol 2. ChCl:ethylene glycol	3	60–150	Lignin Glucose	For 1: Removal of lignin: 33% Glucose yield: 0.80 g/g glucan @150 °C	[179]
Apple residues	1. ChCl:glycerol 2. ChCl:ethylene glycol	3	60–150	Lignin Glucose	For 1: Removal of lignin: 62% Glucose yield: 0.95 g/g glucan @150 °C	[179]
Rice straw	1. ChCl:malic acid 2. ChCl:citric acid 3. ChCl:tartaric acid 4. ChCl:lactic acid 5. ChCl:oxalic acid 6. ChCl:malonic acid 7. ChCl:ethylene glycol 8. ChCl:1,2 propane diol 9: ChCl:urea 10. ChCl:glycerol	0.5–12	60–121	Lignin Glucose Ethanol	For 4: Lignin removal: 57.2% 10. Glucose yield: 87.1% 10. Ethanol yield: 89.5%	[180]
Spruce saw dust	1. ChCl:boric acid 2. ChCl:glycerol 3. Betaine:glycerol	24	80	Glucose	< 20% after EH	[181]

^a^ 10% solids

^b^ 30% solids

^c^ 35% solids

### Biotechnical methods

*Biological pretreatment* can be carried by applying fungi, which breaks down lignin (white-rot fungi), cellulose (soft-rot fungi), or hemicelluloses and cellulose (brown-rot fungi). It has not been common to utilise any of these fungi for pretreatment purposes for, e.g. ethanol production. The rate of degradation is slow, which makes them impractical for industrial use. Another disadvantage is loss of material, which potentially could have been utilised for other purposes. Nevertheless, biological pretreatment has been used to improve methane production from lignocellulosic biomass [182]. Akyol et al. point out that some components in lignocellulosic biomass are difficult to degrade employing anaerobic digestion for biogas production. By including an aerobic step prior to the anaerobic digestion step, where *Trametes versicolor* (a white-rot fungi) was applied on the lignocellulosic biomass, the methane yield improved by 10–18%. The optimum residence time for the aerobic pretreatment step was found to be 6 days. The impact of an additional step will have effects on the overall costs, which need to be evaluated through a techno-economic analysis. Biogas production is the result from anaerobic digestion where a consortium of microorganisms is involved. Ali et al. [183] proposed a biological pretreatment of oak sawdust by means of a microbial consortium prior to the biogas production step. The bacterial pretreatment caused a significant reduction of cellulose, hemicelluloses and lignin content (compared with the untreated sawdust) of 35.8, 37.1 and 46.2%, respectively, after 5 days pretreatment. The biological pretreatment enhanced biomethane formation from untreated oak sawdust by 92% after 40 days of anaerobic digestion. *T. versicolor* has also been employed for production of biosurfactants, which belong to a product family of high value from a biorefinery. Lourenço et al. [184] utilised olive-mill waste in a solid-state fermentation system. The biosurfactant was able to reduce the surface tension by up to 34.5 mN m^−1^. Surfactants have a large application area, such as detergents, cosmetics, pulp and paper and many other areas. *Ceriporiopsis subvermispora* is another fungus that have been studied by Vasco-Correa et al. for pretreatment of lignocellulosic biomass [185]. The investigated the effect of fungal pretreatment on four lignocellulosic materials: corn stover, miscanthus, pine and white ash. The pretreatment procedure was carried out for 14 days of incubation time. The feedstock was treated either sterilised or non-sterilised and the results changed with the sterility. Fungal pretreatment of pine, white ash and miscanthus was successful for the first generation of fungi on non-sterile material. However, for subsequent generations only material that had been sterilised showed signs of degradation. Regarding corn stover, fungal pretreatment of non-sterilised material was inefficient. The highest glucose yield after enzymatic hydrolysis was found to be about 37% for first-generation, non-sterile pretreated white ash wood.

In addition to fungi, bacteria exists that have the capability to degrade various lignocellulosic components. Guo et al. tested various genera from bacteria for laccase production and for their hydrolytic capacity on miscanthus [186]. The strains included, for instance, *Bacillus*, *Pseudomonas*, *Exiguobacterium* and *Aeromonas*. Miscanthus was pretreated for 96 h by adding the substrate to the culture medium containing the microorganism. At the end of the pretreatment, the solid material was washed and subjected to enzymatic hydrolysis for 72 h. The result was a lignin removal after pretreatment of about 30–60%. In addition, the increase in glucose release (compared with untreated Miscanthus) was 1.3- to 2.2-fold higher. A maximum of 87% cellulose digestibility after enzymatic hydrolysis was recorded.

A biological method, which is not by itself a pretreatment method, is the consolidated bioprocessing. It still requires some prior pretreatment to be effective. However, it has some interesting properties, such that it combines enzyme production, enzymatic hydrolysis and fermentation in one vessel. The most commonly employed microorganism are strains of *Clostridium thermocellum*, which has been found to be suitable for, e.g. ethanol production. In a study by Kothari et al. [187], five different pure model cellulose substrates were experimentally evaluated for the effect of enzymatic digestion by *C. thermocellum* in comparison with fungal enzyme mixtures. In the study, no pretreatment prior to degradation took place. The results show that digestion using *C. thermocellum* was more or less affected by structural properties of the substrates, while fungal enzymes yielded a glucan conversion that diminished in the following order: milled filter paper > Avicel > Sigmacell > α-cellulose > cotton linter. The study showed that conversion was rather unaffected by cellulose micro-accessibility in contrast to the selected fungal enzyme blend. It needs to be mentioned that the study was carried out at very low solid content, between 0.5 and 5%. In addition, at 5% solid content, the resulting glucan conversion dropped from above 90% to about 20–30%. However, it is necessary to evaluate real lignocellulosic materials to make proper conclusions.

### Grinding/ultrasound/other mechanical methods

In Northern Europe there is a great interest in waste liquid streams from pulp and paper mills. Lamb et al. [188] used ultrasonication, a Fenton-like reaction, or combinations of the two on steam-pretreated birch wood to investigate if a positive impact on biomethane production could be achieved. A Fenton reaction is based on iron salts in combination that in combination with hydrogen peroxide can oxidise organic contaminants, such as inhibitors and toxic substances that can be detrimental to microorganisms. In the study, birch wood was first steam pretreated at 210 °C for 10 min. Subsequently, the STEX material was subjected to ultrasonication at a pH of 4 for 2 h, with or without addition of hydrogen peroxide and FeCl_3_. The results suggest that the combination of a harsh ultrasonication and Fenton-like treatment have a negative impact on the biogas production rate, while a milder treatment caused increased production rate. This resulted in a shorter residence time to reach close to the maximum BMP, which has a large impact on reactor size (or biogas capacity), which is the major capital cost in a biogas plant.

The combination of thermal pretreatment and ultrasonication was also investigated by John et al. [189] for hydrolysis of sweet lime peel. First, thermal pretreatment was performed with addition of sulphuric acid (0.25% v/v) with a solids concentration of 17%. In an autoclave at 121° for 1 h. The solid material after the thermal treatment was then subjected to ultrasound assisted dilute acid hydrolysis employing a design of experiments method to generate the experimental conditions. The design variables were acid concentration, peel concentration, sonication time, temperature and amplitude. The highest reducing sugar yield was about 0.2 g/g pretreated peel, which is equivalent to about 60% yield based on the cellulose content after pretreatment.

Chen et al. [190] compared four different treatment methods on white birch chips to evaluate the effect of thermochemical pretreatment in terms of its composition. They also assessed thermochemical and disk-refining treatment for production of fermentable carbohydrates. In addition, enzymatic hydrolysis was performed to evaluate the yield of sugars after the pretreatment steps. The evaluation was carried out in pilot-scale, where the starting material was 100 kg of birch chips. Sodium hydroxide (5% w/v dry biomass) was added during the thermochemical step, which was carried out at 140 °C for 30 min. Addition of NaOH during the thermal pretreatment was found to reduce the energy consumption in the disk-refining step by up to 86%.

Particle size reduction can be carried out by a process that is common in the pulp industry, mechanical refining, which yields a pulp that is processed further to pulp qualities such as newspaper and paperboard. In an attempt to increase the recovery of carbohydrates and reduce the enzyme addition, Chandra et al. [68] utilised mechanically refined pulp for subsequent steam pretreatment of poplar pulp. The reactivity of lignin decreased during steam pretreatment while the accessibility to the cellulose fraction was improved. Compared with refiner pulp, the steam-pretreated material performed better during enzymatic hydrolysis. The addition of a pre-processing step where poplar chips were allowed to soak in a solution of either sodium sulphite, or sodium sulphite and sodium carbonate, at 60 °C for 16 h prior to steam pretreatment, caused lignin sulphonation as well as cellulose hydrolysis to become enhanced. In addition, most of the carbohydrate fraction retained in the solid fraction, which is in contrast to acid-catalysed steam pretreatment where the hemicelluloses fraction solubilise.

Physical pretreatment does not only refer to grinding or milling operations. A study by Falls et al. [191] explores the effect of “shock treatment”. The method is based on the effect that a sudden shock wave causes on a material, if treated further using, e.g. enzymatic hydrolysis. In the study, Falls et al. tested several lignocellulosic materials, such as bagasse, corn stover, poplar wood, sorghum and switchgrass. One of the goals was to compare ball milling with shock treatment in terms of the glucan enzymatic digestibility of oxidative-lime pretreated (OLP) substrates. For a hydrolysis time of 24 h, shock treatment was more efficient than ball milling for all substrates. With increasing residence time, ball milling was performing better than shock treatment. Compared with only OLP substrates an increase in digestibility was found, resulting in higher glucose formation.

### Combination of pretreatment methods

Combinations of one or more pretreatment methods to improve the pretreatment process may be a possibility to find process designs that will be suitable for enhanced fractionation of the raw material. This could be, for instance, to yield process streams, which are optimised for hemicelluloses, while other streams are optimised for other compounds. However, implementation of several, different pretreatment methods comes with an additional cost, if the methods are dissimilar. Therefore, it would not be advisable to apply widely different pretreatment methods. Nevertheless, pretreatment is commonly preceded by a size-reduction step, which can be regarded as mechanical pretreatment, if the size reduction is thorough. The reverse operational procedure is also a possibility. Chen et al. [190] used hydrothermal treatment at 140 °C for 30 min of white birch chips that had been impregnated with a solution of 5 wt% NaOH. The collected material was further treated in a disk refiner at various gap sizes ranging from 0.15 to 1.00 mm. The refined material was enzymatically hydrolysed for 72 h. The addition of NaOH improved the combined pretreatment process. Compared with a disk-refining step only, the process improved total-reducing sugar yield 6.4 to 42.2% based on the available cellulose, while the specific energy consumption was reduced by 62%. The sugar yield decreased with an increasing disk gap. The suggested process solution is especially attractive in an already existing pulp mill. In a study performed by Huang et al. [192] dilute-alkali assisted ball milling of bagasse and pennisetum was employed, having NaOH concentrations ranging from 0.25% to 4.0%, followed by mild hydrothermal pretreatment at 80 or 100 °C. The highest reducing sugar yield from bagasse (40.75%) was achieved using 4% NaOH at 100 °C for 40 min for bagasse, while 55.74% yield was obtained for pennisetum after treatment at 80° for 60 min. The definition of the obtained yields are somewhat unclear. A similar study was presented by Yu et al. [193], who compared phosphoric acid pretreatment with intense pulverisation of corn stover, which rendered the material more susceptible to enzymatic hydrolysis.

Other combinations of pretreatment methods include a study by Lee et al. [194], employing ionic liquid fractionation, followed by a solid acid saccharification and enzymatic hydrolysis; mild acid pretreatment of eucalyptus, birch or wheat straw followed by an organosolv step utilising ethanol–water mixtures [195]; sequential fractionation of hardwood by a combination of STEX and hydrotropic treatment [196]. Enzymatic hydrolysis of pine from auto-hydrolysis at temperatures ranging from 150 to 200 °C, was used by Rigual et al. in an ionic-liquid pretreatment step [197], which resulted in a digestibility of about 79% of available glucan content.

The combination of pretreatment methods opens up for better fractionation in many cases, if a selective fraction can be employed, such as pre-extraction of hemicelluloses by acid treatment, which could be used for high-value products such as barrier films or hydrogels, followed by an organosolv step for lignin and cellulose recovery [198]. Thus, there are many suggestions for improving the utilisation of the raw materials by combining various pretreatment methods. However, it is critical to find combinations that match each other to avoid additional complications in terms of process design and costs.

## Conclusions

The increasing interest in the utilisation of lignocellulosic materials from agricultural, forest and other plants and residues is shown in the number of publications in the field. The biorefinery concept is advancing from an interesting idea to a promising alternative for many fossil-based products. Already today, several production facilities can be regarded as a kind of biorefinery, e.g. pulp mills, where more than one product is resulting from lignocellulosic materials. However, recovery of even more constituents and production of other types of compounds calls for fractionation methods that may be different from traditional pulping methods.

The huge variation in lignocellulosic materials makes it difficult to find a general process design for all raw materials. The recalcitrance of softwood is much higher than that of most agricultural or herbaceous crops and residues. Therefore, it is difficult to define “the best pretreatment method”. In the end, this depends on the proposed application, and any recommendation must be based on a thorough techno-economic evaluation, where data has been collected from a scale of at least pilot size. Selection of a suitable pretreatment method largely depends on the final application. Nevertheless, steam pretreatment in various forms has shown to be attractive in the first demo plants for 2G ethanol production; however, for more extensive fractionation of lignocellulosic materials, it may be advantageous to employ pretreatment methods that act by solubilisation.

The impressive number of suggested pretreatment methods and publications clearly shows that the research field is very much alive. The great interest in the more recent pretreatment methods, where ionic liquids and deep-eutectic solvents are investigated, contributes to the publication list; however, the large number of studies regarding steam explosion, organosolv, etc. indicates that they still have a very important role to play when planning for future full-scale biorefineries. An indication can be the number of publications in the last 5 years, where about 1500 papers with keywords containing steam pretreatment (explosion) or organosolv were published. A similar search on ionic liquids or deep-eutectic solvents results in a steady increase from almost none in 2010 to about 200 papers a year today. It is likely that the latter will continue to increase with time.

## Data Availability

Not applicable.

## Abbreviations

*L*_N_: standard litre (dry gas volume at 0 °C and 1013 mbar)
VS: volatile solids g kg^−1^
COD: chemical oxygen demand
TRL: technical readiness level
SEM: scanning electron microscopy
MRI: magnetic resonance imaging
NMR: nuclear magnetic resonance
STEX: steam explosion (steam pretreatment)
LHW: liquid hot-water treatment
BMP: biochemical methane potential
EH: enzymatic hydrolysis
SEC: size-exclusion chromatography
FTIR: Fourier-transform infrared spectroscopy
NMR (HSQC): heteronuclear single quantum coherence nuclear magnetic resonance
RTILs: room-temperature ionic liquids
GVL: γ-valerolactone
THFA: tetrahydrofuran
IL: ionic liquid
DES: deep-eutectic solvent
ChCl: choline chloride
GH: guanidine hydrochloride
[Bmim]: 1-butyl-3-methylimidazolium
[HBim][HSO_4_]: 1-butylimidazolium hydrogen sulfate]
[DMBA][HSO_4_]: *N*,*N*-dimethylbutylammonium hydrogen sulfate
[TEA][HSO_4_]: triethylammonium hydrogen sulfate
[EMim]: 1-ethyl-3-methylimidazolium
[DEP]: diethyl phosphate
[C_2_mim][MeO(H)PO_2_]: 1-ethyl-methylimidazolium methylphosphonate
[AMIM]Cl: 1-allyl-3-methylimidazolium chloride
[Py]: pyridinium
[Mim]: 1-methylimidazolium
[Pyrr]: pyrrolidinium
[OAc]: acetate
EG: ethylene glycol
PG: propylene glycol
GLY: glycerin
PTSA: *p*-toluenesulfonic acid
HBA: hydrogen-bond acceptor
HBD: hydrogen-bond donator
DMEAF: *N*,*N*-dimethylethanolammonium formate
DMEAA: *N*,*N*-dimethylethanolammonium acetate
DMEAG: *N*,*N*-dimethylethanolammonium glycolate
DMEAS: *N*,*N*-dimethylethanolammonium succinate
SCB: sugar cane bagasse
